# The Long Pentraxin PTX3 Controls *Klebsiella Pneumoniae* Severe Infection

**DOI:** 10.3389/fimmu.2021.666198

**Published:** 2021-05-20

**Authors:** Fatemeh Asgari, Domenico Supino, Raffaella Parente, Nadia Polentarutti, Matteo Stravalaci, Remi Porte, Fabio Pasqualini, Marialuisa Barbagallo, Chiara Perucchini, Camilla Recordati, Elena Magrini, Andrea Mariancini, Federica Riva, Alessia Giordano, Sadaf Davoudian, Thierry Roger, Cornelis van’t Veer, Sebastien Jaillon, Alberto Mantovani, Andrea Doni, Cecilia Garlanda

**Affiliations:** ^1^ Department of Inflammation and Immunology, IRCCS Humanitas Research Hospital, Rozzano, Italy; ^2^ Department of Biomedical Sciences, Humanitas University, Pieve Emanuele, Italy; ^3^ Department of Veterinary Medicine, University of Milano, Lodi, Italy; ^4^ Infectious Diseases Service, Department of Medicine, Lausanne University Hospital and University of Lausanne, Epalinges, Switzerland; ^5^ Center of Experimental and Molecular Medicine, Academic Medical Center, Amsterdam, Netherlands; ^6^ Centre for Experimental Medicine and Rheumatology, William Harvey Research Institute, Barts and The London School of Medicine and Dentistry, Queen Mary University of London, London, United Kingdom; ^7^ Unit of Advanced Optical Microscopy, IRCCS Humanitas Research Hospital, Rozzano, Italy

**Keywords:** Pentraxin 3 (PTX3), *Klebsiella pneumoniae*, sepsis, innate immunity, inflammation, complement – immunological term

## Abstract

*Klebsiella pneumoniae* is a common pathogen in human sepsis. The emergence of multidrug-resistant *K. pneumoniae* strains represents a major clinical challenge in nosocomial and community acquired infections. The long pentraxin PTX3, a key component of humoral innate immunity, is involved in resistance to selected pathogens by promoting opsonophagocytosis. We investigated the relevance of PTX3 in innate immunity against *K. pneumoniae* infections using *Ptx3* ^-/-^ mice and mouse models of severe *K. pneumoniae* infections. Local and systemic PTX3 expression was induced following *K. pneumoniae* pulmonary infection, in association with the up-regulation of TNF-α and IL-1β. PTX3 deficiency in mice was associated with higher bacterial burden and mortality, release of pro-inflammatory cytokines as well as IL-10 in the lung and systemically. The analysis of the mechanisms responsible of PTX3-dependent control of *K. pneumoniae* infection revealed that PTX3 did not interact with *K. pneumoniae*, or promote opsonophagocytosis. The comparison of susceptibility of wild-type, *Ptx3^-/-^, C3^-/-^* and *Ptx3^-/-^*/*C3^-/-^* mice to the infection showed that PTX3 acted in a complement-independent manner. Lung histopathological analysis showed more severe lesions in *Ptx3* ^-/-^ mice with fibrinosuppurative, necrotizing and haemorrhagic bronchopneumonia, associated with increased fibrin deposition in the lung and circulating fibrinogen consumption. These findings indicate that PTX3 contributes to the control of *K. pneumoniae* infection by modulating inflammatory responses and tissue damage. Thus, this study emphasizes the relevance of the role of PTX3 as regulator of inflammation and orchestrator of tissue repair in innate responses to infections.

## Introduction

*Klebsiella pneumoniae* is a Gram-negative bacillus belonging to the family of Enterobacteriaceae, normally colonizing the colon and the oropharynx in humans. *K. pneumoniae* is often involved in pneumonia, urinary tract infections, bacteremia and sepsis, in particular in neonates, elderly and immunocompromised patients (1). Overuse of antibiotics has promoted the emergence of multidrug-resistant *K. pneumoniae* strains, which are frequently isolated in nosocomial and community-acquired infections (2, 3). Community-acquired pneumonia (CAP) is the most common cause of sepsis and *K. pneumoniae* is one of the known causative pathogens for CAP with high mortality rates (1, 4, 5).

Sepsis is defined as a life-threatening organ dysfunction caused by a dysregulated host response to an infection with the worldwide mortality rate of almost 11 million individuals per year (6–8). The balance between pro and anti-inflammatory responses contributes to the patient outcome in sepsis (9). The pro-inflammatory responses are associated with cardiovascular disorders, shock, and organ failure, and the anti-inflammatory responses and immune suppression with unpaired control of infections (10–12). In addition to all the complications that sepsis *per se* brings in clinical setting, the emergence of antibiotic resistant *K. pneumoniae* strains becomes a notable concern in public health.

The innate immune system is the first line of defense against invading pathogens and tissue damage. It is composed of a cellular and a humoral arm. The humoral arm comprises the complement system and soluble pattern recognition molecules (PRMs), such as pentraxins, collectins and ficolins (13). Pentraxin 3 (PTX3) is the first member and prototype of the long pentraxin subfamily (14, 15). It is produced locally in damaged tissues by a variety of cells, in particular stromal and immune cells, after stimulation by inflammatory cytokines, such as IL-1β and TNF-α, TLR agonists and other microbial components, such as lipopolysaccharide (LPS), outer membrane protein A (OmpA) and lipoarabinomannans, or intact microorganisms (13, 16).

PTX3 is a multifunctional protein involved in a wide range of activities, ranging from microbial recognition to regulation of inflammation and tissue remodeling. In particular, PTX3 is involved in resistance to selected pathogens, acting as an opsonin, promoting phagocytosis in a Fcγ receptor and complement-dependent manner, amplifying the responses to bacteria and neutralizing viruses (17–20). PTX3 has also been shown to regulate inflammation due to infections (16, 21, 22) or sterile tissue damage (23–25), by modulating the complement cascade and neutrophil recruitment (17). Finally, PTX3 is involved in tissue remodeling, by contributing to fibrinolysis, angiogenesis, matrix deposition (20). PTX3 has been reported to play a dual role in *K. pneumoniae* infections in transgenic mice, depending on the bacterial load and on the amount of overexpressed PTX3 (26). In addition, PTX3 has been shown to contribute to innate immune responses against the outer membrane protein A from *K. pneumoniae* (KpOmpA) (27). Nevertheless, the role of PTX3 and the mechanism of its action in innate immunity against *K. pneumoniae* infections are not clearly defined.

In this study, taking advantage of PTX3-deficient mice and well-established *in vivo* models of *K. pneumoniae* severe infection, we demonstrated the actual protective role of PTX3 in defense against *K. pneumoniae.*

## Material and Methods

### Animals

All mice used were 8-12 weeks old male on a C57BL/6J genetic background. C57BL/6J wild-type mice were purchased from Charles River Laboratories, Calco, Italy. PTX3-deficient mice (*Ptx3^-/-^*) were on a C57BL/6J background, generated as described (28) and bred by Charles River Laboratories, Calco, Italy. C3-deficient mice were from The Jackson Laboratories, Bar Harbour, Maine, US. PTX3/C3-double deficient mice were generated by crossing PTX3- and C3- deficient mice. For the generation of bone marrow chimeras*, Ptx3^-/-^* and wild-type mice were lethally irradiated with a total dose of 900 cGy. Two h later, mice were injected in the retro-orbital plexus with 5x10^6^ bone marrow cells obtained by flushing of the cavity of femurs from wild-type or *Ptx3^-/-^* donor mice. Recipient mice received gentamycin (0.8 mg/ml in drinking water) starting 10 days before and for two weeks after irradiation and used 12 weeks later. For the generation of endothelial conditional PTX3 deficient mice, we used the tamoxifen inducible *cre*/*lox* site-specific recombination system: *Ptx3* ^fl/fl^ *Cdh5*-cre/ERT2*^+/-^* mice were generated crossing mice carrying a loxP-flanked *Ptx3* gene generated by Oxgene with Cdh5-Cre/ERT2*^+/-^* mice from Taconic. Tamoxifen was injected ip for five days (2 mg/mice) to activate cre recombinase in endothelial cells.

Experiments were performed in the animal facility of Humanitas Research Hospital in individually ventilated cages. Mice were randomized based on sex, age, and weight.

### *K. Pneumoniae* Infection Models

Pneumonia and peritonitis were induced by intranasal inoculation with 1x10^4^ CFUs or intraperitoneal injection with 500 CFUs *K. pneumoniae* serotype 2 (ATCC 43816), respectively. Mice were sacrificed at different time points (6, 18, 24, 44 hours) after infection for sample collection. In the survival study, mice were observed 2 times a day for clinical sign of distress and mortality, and sacrificed by humanized euthanasia when they reached the human endpoints indicated by clinical signs (body temperature dropping, intermittent respiration, solitude presence, hunched posture, fur erection, unresponsive alertness, and inability to ascend when induced).

In selected experiments, mice were treated intraperitoneally with 10 μg recombinant mouse-PTX3, produced as previously described (29), with one dose at 24 hours or two doses at 0 and 24 hours after infection, and the outcome of infection was evaluated by following mortality (one treatment) and counting lung and spleen CFU (two treatments).

### Sample Collection

Lung, liver and spleen were collected and homogenized in 1 ml PBS containing protease inhibitors (Complete tablets EDTA-free, Roche Diagnostic) and 1 mM PMSF (Sigma) and used for CFU count. The homogenates were then centrifuged at 13000 rpm for 20 minutes at 4°C and the supernatants were stored at -80°C. Blood was drawn either from the cava vein or the retro-orbital plexus, collected in an appropriate anti-coagulant (heparin, EDTA or citrate) vacutainer tube.

Lung, liver and spleen homogenates and blood were serially diluted 1:10 in PBS, plated in Tryptic Soy Broth (TSB) agar (BD) and incubated overnight at 37°C for CFU count. EDTA-blood samples from retro-orbital plexus were analyzed by the laser automated counter ADVIA 120 (Siemens).

Citrate-blood from cava vein was used to measure fibrinogen by converting the clotting time under bovine thrombin activation (Futurlab srl, Limena, PD Italy). ELISA assays were used to measure murine cytokines, MPO, PTX3 (R&D DuoSet ELISA Development System, USA) and TAT (Thrombin-Antithrombin Complexes- Abcam, Cambridge, UK) according to manufacturer’s instructions. Magnetic beads multiplex immunoassay (ProcartaPlex- Invitrogen) was used to measure cytokines in the blood. Protein quantitation was performed on the Luminex instrument platform (Bio-plex 200 system- Bio-Rad).

### Flow Cytometry Analysis of Leukocyte Populations

Analysis of leukocyte populations in lung and blood was performed by flow cytometry. Representative gating strategy is shown in **Supplementary Figure S1A**. The following murine antibodies were used: Anti-mouse CD11b-BV785 (Clone M1/70) and anti-mouse F4/80-PE-Cy7 (Clone BM8) from Biolegend, anti-mouse Ly6G-PE-CF594 (Clone 1A8), anti-mouse CD45-BV605 (Clone 30-F11), anti-mouse CD11b-APC-Cy7 (Clone M1/70), anti-mouse Ly6C-BV421 (Clone AL-21), anti-mouse CD11c-AF700 (Clone HL3), anti-mouse MHCII-FITC (Clone 2G9) and anti-mouse Siglec-F-PE (Clone E50-2440) from BD, and anti-mouse CD103-PerCP (Clone 2E7) from eBioscience. Briefly, lungs were cut into small pieces with razor blades and digested for 20 min at 37 °C in PBS containing 0.1 mg/ml collagenase A (Roche). Then the suspension was smashed and passed through a cell strainer (70 μm pore size). RPMI (Gibco) containing 10% FBS was added to stop the digestion process. Lung and blood total cells were counted on Neubauer chamber. Cell viability was determined by LIVE/DEAD™ Fixable Aqua Stain Kit (Invitrogen) and CD16/32 Fc block (Clone 93- eBioscience) was used to block non-specific Fc receptor binding. Samples were incubated with the mixture of the antibodies and red blood cells were lysed by adding ice-cold ammonium*-*chloride*-*potassium *(*ACK*)* lysing buffer (0.15 M NH_4_Cl, 10 mM KHCO_3_, 0.1 mM EDTA, pH 7.2). Samples were fixed with 1% paraformaldehyde (PFA) and analyzed on LSR Fortessa (BD Bioscience). Data were analyzed with FlowJo software 10 (Treestar).

### Histological Examination

Mouse organs were fixed in 4% neutral buffered formalin. Paraffin embedded mouse tissues were cut at 3 μm and sections were stained by hematoxylin-eosin. Slides were examined blindly under light microscopy by a veterinary pathologist (C.R.).

### Confocal Microscopy

Lung tissues were frozen in OCT and cut at 8μm. Sections were fixed with 4% PFA and blocked with 2% BSA in PBS^2+^. Sections were then incubated with the following primary antibodies for 2 h at room temperature: affinity purified goat IgG anti-PTX3/TSG-14 (5 µg/ml; R&D Systems), rabbit polyclonal anti-fibrin(ogen) (5 µg/ml; Dako), mouse monoclonal anti–α-SMA FITC (4 µg/ml; Sigma-Aldrich); rat monoclonal anti-CD31 APC (0.5 µg/ml; BD); and rat monoclonal anti-PDGFRα (CD140a) BV421 (5 µg/ml; BD). Sections were then incubated for 1 h with Alexa Fluor (488, 568, or 647)–conjugated species-specific cross-adsorbed detection antibodies (2 µg/ml; Invitrogen). 4’,6-diamidino-2-phenylindole-DAPI (1:1000; Invitrogen) was used to visualize the nucleus. Working solution of 5% of donkey serum (Sigma-Aldrich), 2% BSA, 0.1% Triton X-100 (Sigma-Aldrich) in PBS^2+^, pH 7.4, was used for antibody dilution and sections were washed after each step with washing buffer (PBS^2+^, pH 7.4, containing 0.01% (vol/vol) Tween 20). Lungs from *Ptx3^−/−^* mice were used as negative control for immunostaining of PTX3. The slides were then dried, and coverslips were mounted with fluorsave reagent (Calbiochem, Meudon, France). Samples were analyzed with a laser scanning confocal microscope (FluoView FV1000; Olympus) by excitation at 405, 488, 559, and 635 nm and collection of the emission using at 425–475 nm (for DAPI), 416-458 nm (for BV421), 490–530 nm (for Alexa Fluor 488), 570–615 nm (for Alexa Fluor 568), and 655–700 nm (for Alexa Fluor 647) band-pass filters. Three images/mouse (1,024 x 1,024 pixels) were acquired with 20x objective (20x 0.75 NA Plan-Apochromat; Olympus).

### *In Vitro* Phagocytosis in Murine Whole Blood

*K. pneumoniae* bacteria were labelled using 10 µM Vybrant™ CFDA, SE Cell Tracer Kit (V12883-Thermo Fisher), incubating for 1 hour at room temperature, followed by 3 times washing with HBSS (Hank’s Balanced Salt Solution) and resuspend in PBS^2+^. Efficiency of labeling was measured by flow cytometry. Phagocytosis of *K. pneumoniae* was assessed using whole blood from wild-type and *Ptx3^-/-^* mice, collected in heparin vacutainer. Briefly, 4x10^6^ CFSE-labelled *K. pneumoniae*, either pre-opsonized (85 μg/ml) or not with recombinant human PTX3, were added to 70 μl of whole blood to reach the final concentration of 25 μg/ml and incubated for 20 min at 37°C. Phagocytosis was blocked by placing samples on ice and red blood cells were lysed by adding ice-cold ACK lysing buffer. The samples were then stained by a combination of anti-mouse Ly6G- PECF594, anti-mouse CD45- BV605, anti-mouse CD11b- APC-Cy7, anti-mouse Ly6C- BV421. Samples were fixed with 1% PFA and analyzed by flow cytometry (LSR Fortessa). Representative neutrophil gating strategy is shown in **Supplementary Figure S1B**.

### PTX3 Binding to *K. Pneumoniae*

Binding of PTX3 to *K. pneumoniae* was assessed by flow cytometry and wild-field microscopy. Polyclonal rabbit anti-hPTX3 antibody (29) was biotinylated with NHS-LC-Biotin (Thermo Scientific, Waltham, USA), according to the manufacturer’s instructions. 10^6^ CFSE-labelled *K. pneumoniae* were incubated with PTX3 (ranging from 1 to 20 μg/ml) in PBS^2+^ containing 0.2% (w/v) BSA for 1h at room temperature. Unbound PTX3 was removed by washing. The samples were then incubated with anti-hPTX3 biotinylated polyclonal antibody (0.5 μg/ml) followed by incubation with Streptavidin-Alexa Fluor 647 (Invitrogen™) (1:500) to detect bound proteins. Samples were fixed with 1% PFA and analyzed either on a FACS Canto II flow cytometry system (BD Biosciences, East Rutherford, NJ) or wild-field microscopy. FITC-labeled *Aspergillus fumigatus* conidia were used as positive control (28).

### Production of PTX3 by Fibroblasts

Mouse embryonic fibroblasts (MEF) were obtained from a wild-type pregnant mouse at 14 day post-coitum as previously described (30). Cells were plated at a density of 10^5^/ml and expanded in 6-well plates in RPMI 1640 medium supplemented with 2 mM L-glutamine, 10% FCS, and B-mercaptoethanol. MEF (5x10^5^ cells/well) were plated in a 24-well plate at passage 5-6 in RPMI 1640 medium supplemented with 2 mM L-glutamine, 1% FCS and stimulated with heat-killed *K. pneumoniae* (30 min at 70°C) at ratios of 5:1, 1:1 and 1:5 (bacteria:cells). LPS (10ng/ml) and TNFα (10ng/ml) were used as controls of PTX3 induction. Murine PTX3 concentration in supernatants was analyzed by ELISA (R&D DuoSet ELISA Development System, USA).

### Statistical Analysis

Values were expressed as mean ± SEM or median of biological replicates, as specified. Two-tailed Mann-Whitney test or ANOVA multiple analysis were used. Survival curves are shown as Kaplan-Meier plots and compared using log-rank test. Linear regression analysis (best-fit curve) was used to analyze correlation between PTX3 and cytokine levels. A ROUT test was applied to exclude outliers. The analyses were done using GraphPad Prism 6 (San Diego, CA). *P* < 0.05 was considered statistically significant.

## Results

### PTX3 Expression Is Induced During *K. Pneumoniae* Infection

**Figure 1:**
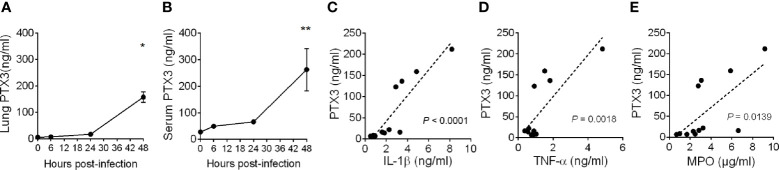
Induction of PTX3 expression during *Klebsiella pneumoniae* infection. PTX3 concentration in lung homogenate **(A)** and serum **(B)** of wild-type mice infected intranasally with 10^4^ CFUs *K. pneumoniae* serotype 2 (ATCC 43816). Each dot is mean ± SEM (n = 4 mice). PTX3 concentration at 6, 24 and 48 hours after infection were compared with basal levels using ANOVA multiple analysis. *6h vs 48h, **0h vs 48h. **(C–E)** Correlation between the concentration of PTX3 and IL-1β **(C)**, TNF-α **(D)** or MPO **(E)** in the lung during the course of the infection. Linear best-fit curve.

Pneumonia was induced in C57BL/6J mice by intranasal inoculation with *K. pneumoniae* serotype 2 (ATCC 43816). Intranasal administration of *K. pneumoniae* leads to a gradually localized infection in the lung followed by systemic dissemination of bacteria, along with inflammatory responses, organ injury, and eventually death, mimicking sepsis (31, 32). To investigate the relevance of PTX3 in this model, we first measured the local and systemic levels of PTX3 during the infection and we observed robust induction of PTX3 starting from 24 hours post-infection and reaching a peak at 48h post-infection, following systemic dissemination, as indicated by spleen bacterial load (**Figures 1A, B** and **Supplementary Figure S2A, B**).

PTX3 expression is induced by both microbial components, including KpOmpA (27), and proinflammatory cytokines, such as TNF-α and IL-1β, and rapidly released by activated neutrophils (16, 33). We thus analyzed the local and systemic concentration of these inflammatory mediators and of MPO, a marker of myeloid cell infiltration and activation. As shown in **Supplementary Figures S3A–F**, we observed an increase of the concentration of IL-1β, TNF-α and MPO during the progression of the infection, which positively correlated with the concentration of PTX3 (*P* < 0.0001, *P* = 0.0018, *P* = 0.0139, respectively) (**Figures 1C–E**). These results suggest an association between *K. pneumoniae* infection-induced inflammation and PTX3 expression.

### PTX3 Deficiency Is Associated With Higher Susceptibility to *K. Pneumoniae* Infection

**Figure 2:**
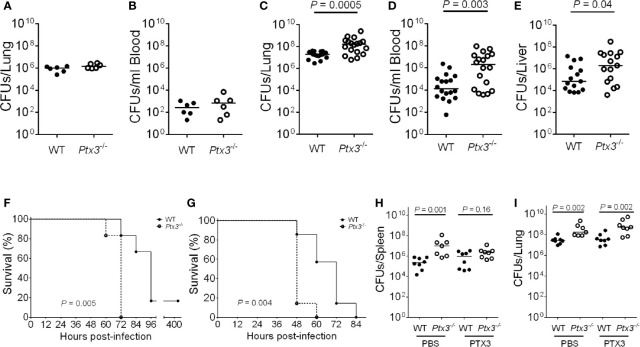
Susceptibility of *Ptx3^-/-^* mice to *K. pneumoniae* infection. **(A–E)** Bacterial load in the lung **(A)** and blood **(B)** at 18 hours after intranasal infection (n = 6 mice) and in the lung **(C)**, blood **(D)**, and liver **(E)**, at 44 hours after intranasal infection (n = 15-18). The median of CFU count is shown. Two-tailed Mann-Whitney test. **(F, G)** Kaplan-Meier survival curves of wild-type and *Ptx3^−/−^* mice inoculated either intranasally with 10^4^ CFUs (n = 6 mice) **(F)** or intraperitoneally with 500 CFUs (n = 7 mice) *K. pneumoniae* **(G)**. Survival curves were compared with the Log-Rank test. **(H, I)** CFU count in the spleen **(H)** and lung **(I)** at 44 hours after intranasal infection in mice treated with mPTX3 (10 μg/mouse at 0 and 24 hours after infection) (n = 7-8 mice). The median of CFU count is shown. Two-tailed Mann-Whitney test. **(A, F)** show one representative experiment out of two **(A, F)**. **(C–E)** are the pool of two separate experiments. **(B, G–I)**; one experiment performed.

*Ptx3* ^-/-^ mice were used to address if PTX3 is involved in the defense against *K. pneumoniae* infection. Early (18 h) after intranasal infection, lung and blood bacterial loads were comparable in *Ptx3^+/+^* and *Ptx3* ^-/-^ mice (**Figures 2A, B**). In contrast, at the late stage of infection (44 h), PTX3-deficiency was associated with more severe local and systemic infection. In particular, bacterial loads were 15-fold higher in the lungs (*P* = 0.0005), 55-fold higher in the blood (*P* = 0.003), 14-fold higher in the liver (*P* = 0.04) of *Ptx3* ^-/-^ mice (**Figures 2C–E**). These results indicate that PTX3 is involved in controlling *K. pneumoniae* burden and dissemination at the late stage of infection.

To assess the importance of PTX3 in the outcome of *K. pneumoniae* infection, the mortality rate was investigated using two different models of infection. Wild-type and *Ptx3* ^-/-^ mice were inoculated either intranasally or intraperitoneally with *K. pneumoniae* and observed for 5 days. *Ptx3* ^-/-^ mice showed a higher mortality rate compared to wild-type mice in both models of infection, with 100% *Ptx3* ^-/-^ mice dead compared to 17% wild-type mice 72 h post intranasal infection (*P* = 0.005), and 100% *Ptx3* ^-/-^ mice dead compared to 42% wild-type mice 60 h post intraperitoneal infection (*P* = 0.004) (**Figures 2F, G**). Hence, PTX3 deficiency is associated with worst outcome in *K. pneumoniae* infections.

Finally, we investigated the specificity of the phenotype of *Ptx3* ^-/-^ mice in pneumosepsis induced by *K. pneumoniae* by treating them with recombinant PTX3. Recombinant mouse PTX3 (mPTX3) was administered at the time of infection and 24 h later in wild-type and *Ptx3* ^-/-^ mice. With this schedule of treatment, we observed a better control of the infection in *Ptx3* ^-/-^ mice, since PTX3 treatment abolished the difference in spleen CFUs between *Ptx3* ^-/-^ and wild-type mice (**Figure 2H**). In contrast, the treatment did not improve the control of lung infection in either group nor systemic infection in wild-type mice (**Figures 2H, I**). In addition, in one experiment of survival, the increased mortality of *Ptx3* ^-/-^ mice compared to wild-type mice was abolished in treated mice (P=0.039 in untreated vs P=0.27 in treated mice, n=7, not shown).

### PTX3 Promotes Innate Resistance to *K. Pneumoniae* Infection in an Opsonophagocytosis and Complement-Independent Manner

**Figure 3:**
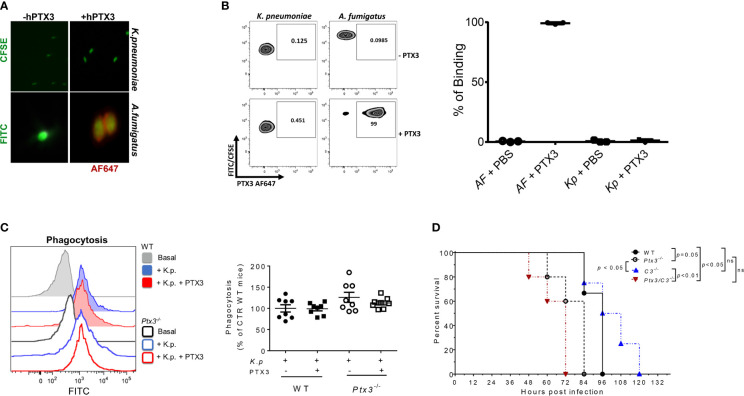
Analysis of opsonophagocytosis and complement in PTX3-dependent resistance to *K. pneumoniae*. **(A)** Immunofluorescence analysis by wild-field microscopy and **(B)** flow cytometry analysis of CFSE-labelled *K. pneumoniae* (upper panels in **(A)**, left zebra plot panels in **(B)** and FITC- labeled *A. fumigatus* (lower panels in **(A)** and right zebra plot panels in **(B)** incubated with or without PTX3, followed by anti-hPTX3 biotinylated antibody and streptavidin-Alexa Fluor 647 (AF647). Quantification of percentage of binding of hPTX3 (20 μg/ml) to *K. pneumoniae* (Kp) and *A. fumigatus* (AF) of the three experiments performed is shown in the right panel in **(B)** FITC-labelled *A. fumigatus* was used as positive control. **(C)** Left panel: Representative histogram showing phagocytosis (CFSE fluorescent intensity in CD11b^+^Ly6G^+^ neutrophils) in wild-type or *Ptx3^-/-^* blood. Basal = blood without *K. pneumoniae*. Right panel: Phagocytosis of CFSE-labelled (*K*) *pneumoniae* by wild-type or *Ptx3^-/-^* neutrophils with or without PTX3 (25 μg/ml). Results are reported as a percentage of wild-type neutrophil phagocytosis (n = 8 mice, from two separate experiments). Graphs show Mean ± SEM. Two-tailed Mann Withney test and ANOVA. **(D)** Kaplan-Meier survival curve of wild-type, *Ptx3* ^-/-^, C3^-/-^ and *Ptx3/C3^-/-^* mice inoculated intranasally with *K. pneumoniae*. N= 3 wild-type, 5 *Ptx3^-/-^*, 4 *C3^-/-^* and 5 *Ptx3/C3^-/-^* mice. Survival curves were compared with the Log-Rank test.

PTX3 was previously shown to be involved in innate resistance to specific infections by opsonizing microorganisms (e.g. *A. fumigatus* conidia and *Pseudomonas aeruginosa*), directly (15, 28, 34, 35) or through ficolins and collectins (13, 36–38), and promoting complement-dependent phagocytosis. Previous studies showed that PTX3 did not interact with *K. pneumoniae*, strain ATCC-27736 (26), even though it bound to purified KpOmpA, cooperating with TLR2 and scavenger receptors in the activation of innate immune responses to this microbial moiety (27). To assess the mechanisms responsible for the protective role of PTX3 in *K*. *pneumoniae* infections, we further investigated a potential interaction between PTX3 and *K*. *pneumoniae*. The binding of PTX3 to *K*. *pneumoniae* was assessed using wild-field microscopy and flow cytometry. We did not observe any interaction with either approach (**Figures 3A, B**). In contrast and as expected, PTX3 interacted with *A. fumigatus* conidia, which were used as positive control in these assays.

To investigate whether PTX3 improved the phagocytic activity, a phagocytosis assay was performed in whole blood drawn from wild-type and *Ptx3* ^-/-^ mice and in the presence of recombinant PTX3 (25 μg/ml). As shown in **Figure 3C**, there were no significant differences in phagocytosis between wild-type and *Ptx3* ^-/-^ neutrophils and the addition of PTX3 in the assay did not promote it.

Finally, we assessed the contribution of complement in PTX3-mediated activity in *K. pneumoniae* infection, by comparing the survival of wild-type and *Ptx3^-/-^* mice to *C3^-/-^* and *Ptx3^-/-^*/*C3^-/-^* mice after intranasal infection. As shown in **Figure 3D**, *C3^-/-^* mice showed mortality comparable to that of wild-type mice (*P* = 0.9), suggesting that complement does not play a major role in this infection. This result is in line with a previous study showing that the strain of *K. pneumoniae* used in this study (ATCC43816) is resistant to complement killing activity (39). In addition, *C3^-/-^* mice showed a lower mortality rate than *Ptx3^-/-^*and* Ptx3*/*C3^-/-^* mice (*P* < 0.05 and *P* < 0.01, respectively). Finally, no significant difference was observed between *Ptx3^-/-^*and* Ptx3*/*C3^-/-^* mice, indicating that the phenotype of PTX3-deficient mice did not depend on complement activation or modulation.

### PTX3 Deficiency Is Associated With Exacerbated Sepsis-Induced Inflammation and Tissue Damage

**Figure 4:**
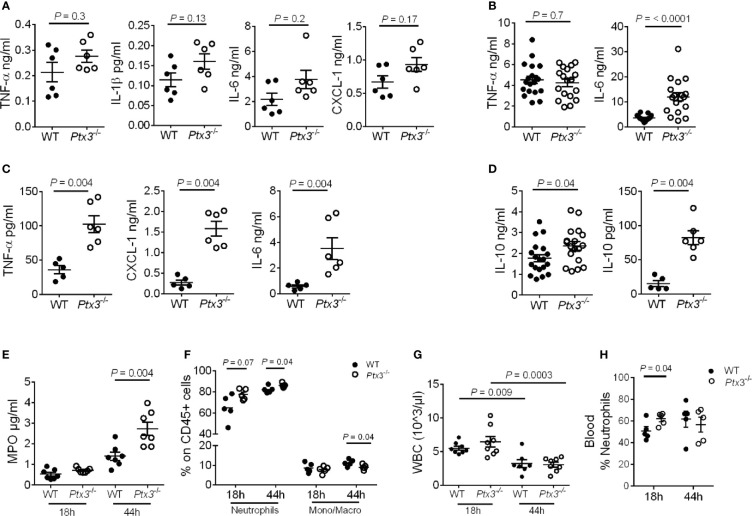
Analysis of inflammation in (*K.*) *pneumoniae* infection. **(A-C)** Cytokine concentration in lung homogenates at 18 hours (n = 6 mice) **(A)** and 44 hours (n = 18 mice, pool of two separate experiments) post-intranasal infection **(B)** or in blood 44 hours post-intranasal infection (n = 5-6 mice) **(C)**. **(D)** IL-10 concentration in lung homogenate (n = 18 mice, pool of two separate experiments) and blood (n = 5-6 mice) 44 hours post-intranasal infection. **(E)** MPO activity in lung homogenates (n = 7 mice). **(F)** Flow cytometry analysis of neutrophils and monocytes/macrophages in the lung (n = 5 mice). **(G)** White blood cell count (n = 7-8 mice). **(H)** Percentage of neutrophils in blood (n = 5 mice). Mean ± SEM is shown. **(A–D, F, H)** Mann Whitney test. **(G)** Multiple *t*-test. The outliers were removed through ROUT.

We next evaluated local and systemic inflammation at early and late time points post-infection. As shown in **Figures 4A, B**, significantly higher IL-6 concentration was observed at a late time point (mean ± SEM: 3.6 ± 0.3 ng/ml in WT and 12 ± 1.7 ng/ml in *Ptx3^-/-^, P* < 0.0001), whereas only a trend of increased concentration of IL-1β, TNF-α, IL-6 and CXCL1, a key neutrophil chemotactic factor, was observed at the early time point in lung homogenate of *Ptx3^-/-^* mice. We observed an exacerbated systemic inflammatory response in *Ptx3^-/-^* mice at the late time point, with significantly higher plasma concentration of TNF-α (36.2 ± 6.2 pg/ml in WT and 102.7 ± 12.3 pg/ml in *Ptx3^-/-^*, *P* = 0.004), IL-6 (0.6 ± 0.1 ng/ml in WT and 3.5 ± 0.8 ng/ml in *Ptx3^-/-^*, *P* = 0.004), and CXCL1 (0.3 ± 0.06 ng/ml in WT and 1.6 ± 0.2 ng/ml in *Ptx3^-/-^*, *P* = 0.004) (**Figure 4C**). Interestingly, PTX3-deficiency was also associated with significantly higher local and systemic concentrations of IL-10 at the late time point (1.7 ± 0.2 ng/ml in WT and 2.3 ± 0.2 ng/ml in *Ptx3^-/-^*, *P* = 0.04 in lung homogenate, and 15.3 ± 4.4 pg/ml in WT and 82.3 ± 10.1 pg/ml in *Ptx3^-/-^*, *P* = 0.004 in blood) (**Figure 4D**). These results suggest the coexistence of hyperinflammatory and immunosuppressive responses during pneumosepsis in *Ptx3^-/-^* mice.

In addition, PTX3 deficiency was associated with a higher amount of MPO (*P* = 0.004 at 44h after infection) (**Figure 4E**) and a higher percentage of neutrophils in the lung (*P* = 0.04 at 44 hours after infection) (**Figure 4F**). We next analyzed total peripheral blood leukocytes and, in the context of comparable sepsis-induced leukopenia (**Figure 4G**), neutrophilia appeared already at the early time point after infection in *Ptx3^-/-^* mice (*P* = 0.04) (**Figure 4H**). Since there was not yet a difference in bacterial load in the two groups of mice at this time point, these results suggest that early neutrophilia was not secondary to increased bacterial load, but rather to exacerbated inflammation.

**Figure 5:**
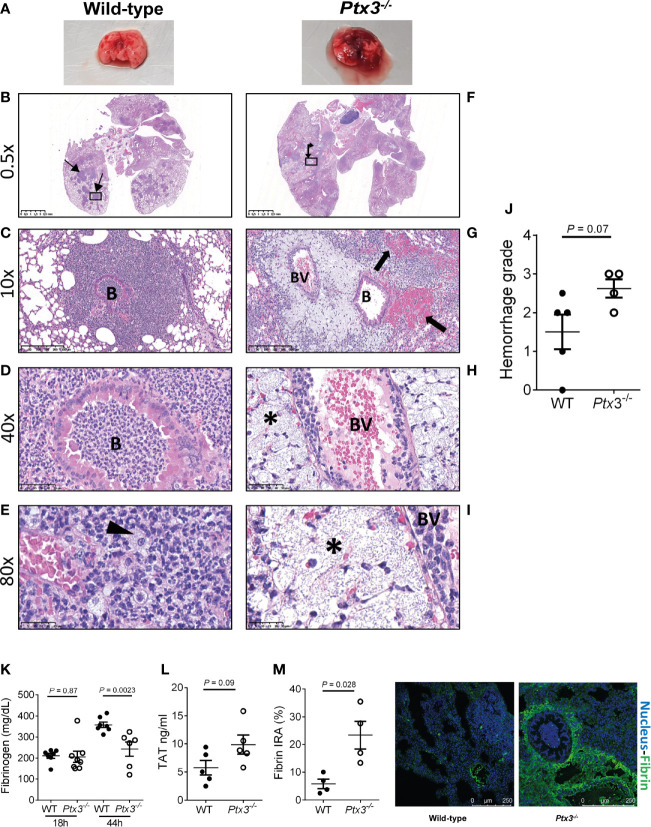
Analysis of lung damage and fibrin deposition of *K. pneumoniae* intranasally infected mice. **(A)** Macroscopic aspect of lungs of wild-type and *Ptx3^-/-^* mice infected with *K. pneumoniae.* A representative picture out of five is shown. **(B–E)**: Histological analysis of wild-type mice. **(B)** Multiple foci of suppurative bronchopneumonia (arrow line). **(C, D)** Lesions centered on bronchiolar structures. **(E)** Bacteria in the bronchiolar and alveolar spaces or within foamy macrophages (arrowhead). **(F–I)**: Histological analysis of *Ptx3^-/-^* mice. **(F)** Multifocal areas of fibrinosuppurative and necrotizing and/or hemorrhagic bronchopneumonia (arrow left-up). **(G)** Intra alveolar and intrabronchial hemorrhages (arrow). **(H, I)** Perivascular aggregates of bacteria associated with edema (*). B, Bronchiole; BV, blood vessel. **(J)** Quantification of the hemorrhage grade in the lung (n = 4 mice). **(K)** Blood fibrinogen concentration (n = 7-8 mice) at 18 and 44 hours after infection and **(L)** TAT levels (n = 5 mice) at 44 hours after infection. **(M)** Fibrin deposition in the lung by confocal microscopy. Left panels: representative images of wild-type and *Ptx3^-/-^* mice. Right panel: Quantitation of fibrin/ogen deposition. Each dot represents the mean percentage of the immunoreactive area (IRA) of four fields per mice (n = 4 mice). **(J–M)** Mean ± SEM is shown. Groups were compared using two-tailed Mann-Whitney test.

We next investigated lung histopathology 44 hours after intranasal infection. First, gross examination of lung tissue showed more severe hemorrhaging pattern and congestion in *Ptx3^-/-^* mice compared to wild type mice (**Figure 5A**). Histopathology showed multiple foci of suppurative bronchopneumonia with intrabronchial and interalveolar bacteria, consistent with *K. pneumoniae* infection, in wild-type mice (**Figures 5B–E**). In contrast, *Ptx3^-/-^* mice developed multifocal areas of fibrinosuppurative and necrotizing and/or hemorrhagic bronchopneumonia with bacteria mainly localized in the perivascular interstitium (**Figures 5F, G**). In wild-type mice, bacteria were not readily visible and were occasionally found in the bronchiolar and alveolar spaces or within foamy macrophages (**Figure 5E**), while in *Ptx3^-/-^* mice perivascular aggregates of bacteria with a mucoid aspect associated with edema were observed (**Figures 5H, I**). Microscopic hemorrhagic lesions were scored, and a higher grade was attributed to *Ptx3^-/-^* mice, but it did not reach the statistical significance (**Figure 5J**). These results suggest that the infection was confined to the bronchial and alveolar spaces in wild-type mice, whereas it spread to the perivascular interstitium and was associated with edema, fibrinosuppurative, and necrotizing and/or hemorrhagic lesions in *Ptx3* ^-/-^ mice.

Fibrinogen plasma concentration increases in inflammatory conditions as part of the acute phase response, but can decrease in sepsis conditions as a consequence of increased consumption due to disseminated intravascular coagulation (DIC) (40, 41). Fibrinogen plasma levels increased during the infection in wild-type mice. At the early time point, fibrinogen levels were comparable in the two groups, but were significantly lower in *Ptx3^-/-^* mice at the late time point compared to wild-type mice (**Figure 5K**). Further, a trend of increase of TAT levels was observed (**Figure 5L**). These results show that despite increased inflammation (**Figure 4**), PTX3-deficiency was associated with lower fibrinogen levels, suggesting DIC and fibrinogen consumption. In addition, immunofluorescence analysis revealed higher fibrin deposition in the lung tissue of *Ptx3^-/-^* mice compared to wild-type mice (4-fold increase of the immunoreactive area, *P* = 0.028) (**Figure 5M**). Finally, the analysis of different organs at 24 hours after intraperitoneal infection indicated an increase in the number of thrombotic lesions in *Ptx3^-/-^* mice when compared to wild-type mice, although it did not reach the statistical significance (**Supplementary Figure S4A–F**).

Collectively, these results suggest that PTX3-deficiency is associated with more severe local and systemic inflammation, tissue damage, as well as signs compatible with DIC and coagulopathy, including increased fibrin deposition in the lungs, which may contribute to increased susceptibility and mortality in this model of pneumosepsis.

### PTX3 Is Produced by Non-Hematopoietic Cells During *Klebsiella Pneumoniae* Infections

**Figure 6:**
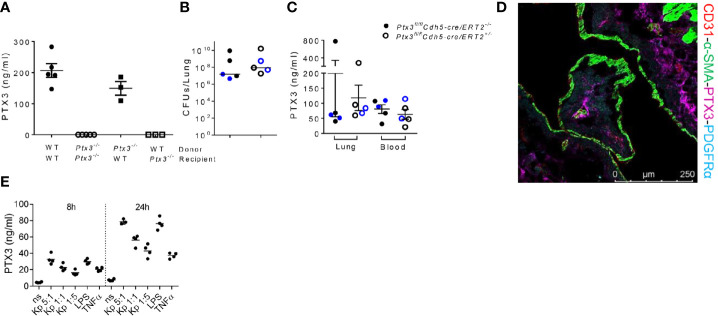
Analysis of the cellular source of PTX3 **(A)** PTX3 protein concentration in serum of chimeric mice 44 hours after infection. Mean ± SEM is shown (n = 3-5 mice). **(B)** Bacterial load in the lung of *Ptx3* ^fl/fl^ *Cdh5*-cre/ERT2^+/-^ and *Ptx3* ^fl/fl^ *Cdh5*-cre/ERT2^-/-^ mice 44 hours after infection. The median of CFU count is shown. **(C)** PTX3 concentration in lung homogenate and blood of *Ptx3* ^fl/fl^ *Cdh5*-cre/ERT2^+/-^ and *Ptx3* ^fl/fl^ *Cdh5*-cre/ERT2^-/-^ mice. Mean ± SEM is shown. n = 5 male and female mice (shown in black and blue, respectively). Groups were compared using two-tailed Mann-Whitney test. **(D)** Immunofluorescence confocal microscopy images of PTX3 (magenta), PDGFRα (blue), CD31 (red) and α-SMA (green) staining in the lung of wild-type mice 44 hours after infection. Representative merged image is shown (n = 3 mice; 3 fields were observed for each mouse). Scale bar = 250μm. **(E)** PTX3 production by embryonic fibroblasts after stimulation with heat killed *K. pneumoniae* at different bacteria:cells ratio for 8 and 24 h. TNF and LPS were used as positive control.

Having observed that PTX3-mediated opsonophagocytosis was not relevant, we further dissected the role of PTX3 in this model by investigated its cell source during *K. pneumoniae* infection. Bone marrow chimeric mice were used to address the involvement of hematopoietic and stromal cells. The evaluation of PTX3 expression during *K. pneumoniae* infection in chimeric mice showed that only the genotype of recipient mice impacted on PTX3 plasma levels of infected mice, indicating that non-hematopoietic cells were a major source of PTX3 expression, whereas hematopoietic cells were irrelevant (**Figure 6A**).

Endothelial cells (ECs) have been known as one of the non-hematopoietic sources of PTX3 (42). To determine whether endothelial cells were the source of PTX3 during *K. pneumoniae* infection, we investigated the expression of PTX3 using tamoxifen-responsive *cdh5*-Cre/lox-dependent conditional *Ptx3* knockout mice (*Ptx3* ^fl/fl^ *Cdh5*-cre/ERT2). As shown in **Figures 6B, C**, we did not observe differences in lung bacterial load (**Figure 6B**) and in local or systemic expression of PTX3 (**Figure 6C**) in *Ptx3* ^fl/fl^ *Cdh5*-cre/ERT2^+/-^ mice compared to *Ptx3* ^fl/fl^ *Cdh5*-cre/ERT2^-/-^. These results indicate that endothelial cells are not the main non-hematopoietic source of PTX3.

We next investigated the localization of PTX3 in the lung by confocal microscopy. In particular, we investigated the association of PTX3 with CD31 (a marker of endothelial cells), α-smooth muscle actin (α-SMA) (a marker of smooth muscle cells), and with platelet-derived growth factor receptor-α (PDGFRα) (a marker of mesenchymal cells). As shown in **Figure 6D**, PTX3 expression was mostly localized in the extracellular space in close proximity of PDGFRα^+^ mesenchymal cells, whereas minimal or no association was observed with endothelial cells or smooth muscle cells, respectively. We finally investigated PTX3 expression by stromal cells after *in vitro* stimulation with *K. pneumoniae*, using embryonic fibroblasts as cellular model for mesenchymal cells. As shown in **Figure 6E**, PTX3 was induced in this cell type in a dose- and time-dependent manner after incubation with heat killed *K. pneumoniae*, as well as by TNF and LPS. These results are in line with previous observations in mouse tissue damage models, such as skin wound healing or liver and lung damage, where PDGFRα^+^ cells were found to be a major source of PTX3 expression (24). Bioinformatic analysis of single cell RNA-seq publicly available datasets confirmed that in addition to vascular endothelial cells, PDGFRα^+^ and PDGFRβ^+^ stromal cells are the major source of PTX3 in mouse and human lung in healthy conditions (43, 44).

## Discussion

The present study was designed to address the role of the long pentraxin PTX3 in innate resistance to *K*. *pneumoniae* infection, taking advantage of PTX3-deficient mice and well-established *in vivo* models of *K. pneumoniae* severe infection. The results obtained demonstrate a clear involvement of PTX3 in the control of the infection, in terms of bacterial load, mortality and inflammation. PTX3 was shown to act through mechanisms independent of opsonophagocytosis or complement regulation. PTX3-dependent regulation of inflammation and tissue damage were found the major mechanisms responsible of the outcome of the infection.

Previous studies showed that PTX3 has a dual role in *K. pneumoniae* infection in PTX3-overexpressing mice, depending on the severity of the inflammatory response induced by the given bacterial load and the amount of protein expressed (26). Overexpression of PTX3 in mice receiving low dose of bacteria promoted the resistance against *K. pneumoniae* respiratory infection, while in mice receiving a high inoculum, overexpression of PTX3 was associated with faster lethality, suggesting the relevance of PTX3-dependent inflammation tuning in this infection (26). In addition, PTX3 was shown to interact with KpOmpA, contributing to the innate response to this microbial moiety (27). However, the effect of PTX3 in the response to *K. pneumoniae* infection could not be inferred by this result. Our study was performed to elucidate the actual role of PTX3 in this infection and its mechanisms of action.

Previous studies showed that PTX3 deficiency is associated with higher bacterial load, more severe outcome and mortality after infection with *A. fumigatus*, *P. aeruginosa, Escherichia coli* and *Shigella flexneri* (22, 28, 45). We obtained similar results upon intranasal or intraperitoneal infection with *K. pneumoniae*, where PTX3 deficiency was associated with higher local and systemic bacterial load and accelerated mortality. These data confirm the role of PTX3 in innate immune responses and resistance to selected pathogens and widen its spectrum of activity in infectious diseases, in particular toward a pathogen frequently involved in human sepsis and associated with multi-drug resistance (2, 4).

The protective role of PTX3 during *A. fumigatus* and *P. aeruginosa* infection was attributed to an opsonic activity followed by enhanced opsonophagocytosis in a complement and Fcγ receptors-dependent manner (15, 28, 34). In this study, we did not observe direct interaction of PTX3 with the K2 strain of *K. pneumoniae*. This suggests that even if PTX3 binds to purified KpOmpA (27), the binding to the bacteria is impaired, possibly by the presence of the capsule. Furthermore, exogenous recombinant PTX3 did not enhance bacterial phagocytosis. These results indicate that the protective role of PTX3 in this model of infection was not mediated by opsophagocytosis, in line with the capacity of *K. pneumoniae* to evade this first line mechanism of defence through the expression of a polysaccharide capsule (2). Resistance to phagocytosis of K1 and K2 *K. pneumoniae* strains by alveolar macrophages and neutrophils has been attributed to various factors, for instance, sialic acid residues which mimic endogenous molecules, and lack of specific mannose residue repeats which prevents efficient lectin-dependent phagocytosis through macrophage mannose receptor and lung-secreted SP-A (2). Therefore, the marginal phagocytosis observed in our experiments could be due to these escaping mechanisms of *K. pneumoniae*, which were not counteracted by PTX3.

Treatment with recombinant PTX3 reduced *P. aeruginosa* colonization in cystic fibrosis mice and synergized with antifungals in the therapy of *A. fumigatus* infections (15, 18, 28, 46), paving the way for the development of PTX3 as potential new therapeutic molecule in these opportunistic infections. In our study, treatment with recombinant PTX3 decreased the systemic spread of the infection and mortality in *Ptx3* ^-/-^ mice, but did not ameliorate the control of the infection in wild-type mice. These results demonstrate that the phenotypes observed were specifically due to PTX3-deficiency and confirm the role of PTX3 in this infection, but further experiments with different treatment schedules would be necessary to demonstrate its therapeutic potential in PTX3-competent individuals.

The crosstalk between PTX3 and the complement system has a dual nature. By interacting with complement recognition molecules, e.g. C1q, ficolins and MBL, PTX3 increases the recognition potential of the humoral innate immunity, leading to increased complement-dependent opsonophagocytosis (13). On the other hand, by binding to factor H or recruiting C4BP, PTX3 tunes activation of complement, preventing excessive activation of complement and inflammation (13, 47). The latter effect has been observed in myocardial infarction and demonstrated in cancer (20, 25, 48–50). By using mice deficient of C3 and mice deficient of both C3 and PTX3, we showed that the complement system does not play a major role during *K. pneumoniae* infection. This is in line with studies showing that either the capsule or LPS can prevent killing by complement of various strains of *K. pneumoniae*. In particular, it has been shown that the K2 strain (ATCC43816) used in our study is protected against killing by human serum (39). In addition, *Ptx3/C3* double deficient mice were not protected compared to *Ptx3* ^-/-^ mice, indicating that PTX3-mediated regulation of complement-dependent inflammation was not involved in the phenotype. Thus, neither complement-dependent opsonophagocytosis nor complement-dependent inflammation were involved in the phenotype of PTX3-deficient mice.

Balanced inflammatory responses contribute to the elimination of infections, whereas exaggerated inflammation can contribute to tissue damage and immunopathology, promoting sepsis (12, 51, 52). In particular, neutrophil-dependent inflammation may contribute to lung immunopathology in pneumonia (35, 53). Through exacerbated release of toxic agents such as proteinases (neutrophil elastase, cathepsin G and proteinase-3), cationic polypeptides, cytokines, ROS, and finally, neutrophil extracellular traps (NETs), neutrophils can contribute to increased epithelial and vascular permeability leading to pulmonary damage, which allow for accelerated transmigration of neutrophils into the alveolar space and bacterial systemic spread (53). During systemic infection, NETs and their content also contribute to endothelial damage promoting diffuse thrombosis, DIC and acute organ injury (54). We observed higher inflammatory responses as well as neutrophil recruitment and activation in *Ptx3^-/-^* mice. In particular, higher neutrophilia was observed in an early time point of infection, when there was still no difference in bacterial load between the two groups of mice, which emphasizes that the higher inflammatory responses in *Ptx3^-/-^* mice is not secondary to higher bacterial load. PTX3 was shown to tune neutrophil recruitment by binding to endothelial P-selectin and following inflammation-induced tissue damage (19). This mechanism has been observed in acute lung injury, pleurisy and mesenteric inflammation, as well as in acute kidney injury (13, 19, 23). Increased MPO levels and neutrophil recruitment in the lung could suggest that P-selectin-dependent deregulated mechanisms account for the phenotypes observed in our model. However, P-selectin deficiency has been associated with higher bacterial burdens and more severe late-stage *K. pneumoniae* pneumosepsis, thus indicating that P-selectin dependent neutrophil recruitment is essential to establish innate immune responses against *K. pneumoniae* (55).

*Ptx3^-/-^* mice developed multifocal areas of fibrinosuppurative and necrotizing and/or haemorrhagic bronchopneumonia, and the infection diffused to the perivascular interstitium, compared with milder tissue damage and confinement of bacteria in the bronchial and alveolar spaces of wild-type mice. Increased fibrin/fibrinogen deposition in the inflamed lung of *Ptx3^-/-^* mice associated with decreased blood fibrinogen levels were also observed. These last phenotypes are in line with previous findings obtained in *Ptx3^-/-^* mice in models of tissue damage, including acute lung injury, where altered thrombotic responses to injury and augmented fibrin deposition were observed (21, 24, 56–59). Furthermore, pharmacologic inhibitors of fibrin deposition rescued the phenotype of PTX3-deficient mice in specific tissue damage models (24). In these contexts, in the acidic environment due to metabolic adaptation during tissue repair, PTX3 interacted with fibrinogen and plasminogen and promoted plasmin-mediated fibrinolysis (24). It was shown by *in vivo* and *in vitro* studies that thrombin improved the host defense against *K. pneumoniae* (ATCC 43816) *via* thrombin-induced fibrin generation and polymerization (60), a finding which is in contrast with ours. On the opposite, our results remind the condition described in human sepsis patients, where activation of coagulation and fibrin deposition are associated with microvascular and multiple organ failure (61), thus suggesting the complexity of the consequences of coagulation and fibrin formation in this infection. Finally, the major cell source of PTX3 during *K. pneumoniae* was represented by stromal cells and not hematopoietic cells. In infected lungs, PTX3 localized in proximity of PDGFRα^+^ mesenchymal cells, further supporting its role in this infection is associated with tissue remodeling and not in pro-phagocytic activity. Further studies will be necessary to assess which of the phenotypes observed in PTX3-deficient mice depend on the lack of the profibrinolytic activity of PTX3.

*PTX3* genetic variants have been associated to innate resistance to infections in humans (62, 63) and PTX3 is a potential therapeutic target in opportunistic infections. Our study clearly demonstrates that PTX3 contributes to the innate resistance to *K. pneumoniae* lung and systemic infections and suggests that a combination of PTX3-mediated mechanisms, which are not related to opsonophagocytosis, concur to the protection. These include regulation of local and systemic inflammation, as well as tuning of immunopathology. Thus, this study emphasizes the relevance of the role of PTX3 as orchestrator of inflammation and tissue repair in innate responses to infections. The identification of endogenous mechanisms of resistance may lead to the early identification of susceptible individuals based on genetic variability and to the development of new therapeutic targets.

## Data Availability Statement

The raw data supporting the conclusions of this article will be made available by the authors, without undue reservation.

## Ethics Statement

Procedures involving animals and their care were conformed with protocols approved by the Humanitas Research Hospital (Milan, Italy) in compliance with national (D.L. N.116, G.U., suppl. 40, 18-2-1992 and N. 26, G.U. March 4, 2014) and international law and policies (EEC Council Directive 2010/63/EU, OJ L 276/33, 22-09-2010; National Institutes of Health Guide for the Care and Use of Laboratory Animals, US National Research Council, 2011). The study was approved by the Italian Ministry of Health (approvals n. 742/2016-PR, issued on 26/07/2016). All efforts were made to minimize the number of animals used and their suffering.

## Author Contributions

CG and AlM contributed to the conception of the study. CG, FA, ReP, SJ, and AD contributed to the design of the study. FA, DS, RaP, NP, MS, ReP, EM, AnM, CR, MB, FR, FP, and CP performed the experiments. FA contributed to the data collection. FA, AD, TR, CV, and CG contributed to the data analysis. FA, DS, SD, and MS performed the statistical analysis. CG and FA wrote the manuscript. All authors contributed to the article and approved the submitted version.

## Funding

The project was funded by the European Commission (European Sepsis Academy Horizon 2020 Marie Skłodowska-Curie Action: Innovative Training Network (MSCA-ESA-ITN, grant number 676129; and European Research Council PHII – 669415 to AM).

## Conflict of Interest

AIM and CG obtain royalties on pentraxin-3 related reagents.

The remaining authors declare that the research was conducted in the absence of any commercial or financial relationships that could be construed as a potential conflict of interest.
