# Supplementary material for: The Long Pentraxin PTX3 Controls Klebsiella Pneumoniae Severe Infection
Source: Front Immunol. 2021 May 20;12:666198. doi: 10.3389/fimmu.2021.666198 (PMC8173212; doi:10.3389/fimmu.2021.666198)
Supplement: Supplementary file 1 [file Table_1.docx]

**Supplementary Figures**

(A)

A

**
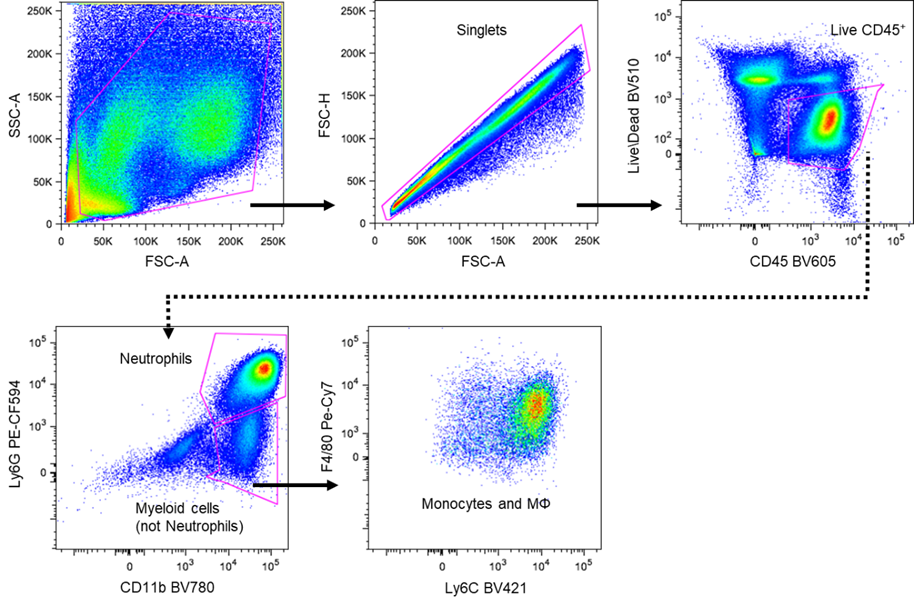
**

B

**
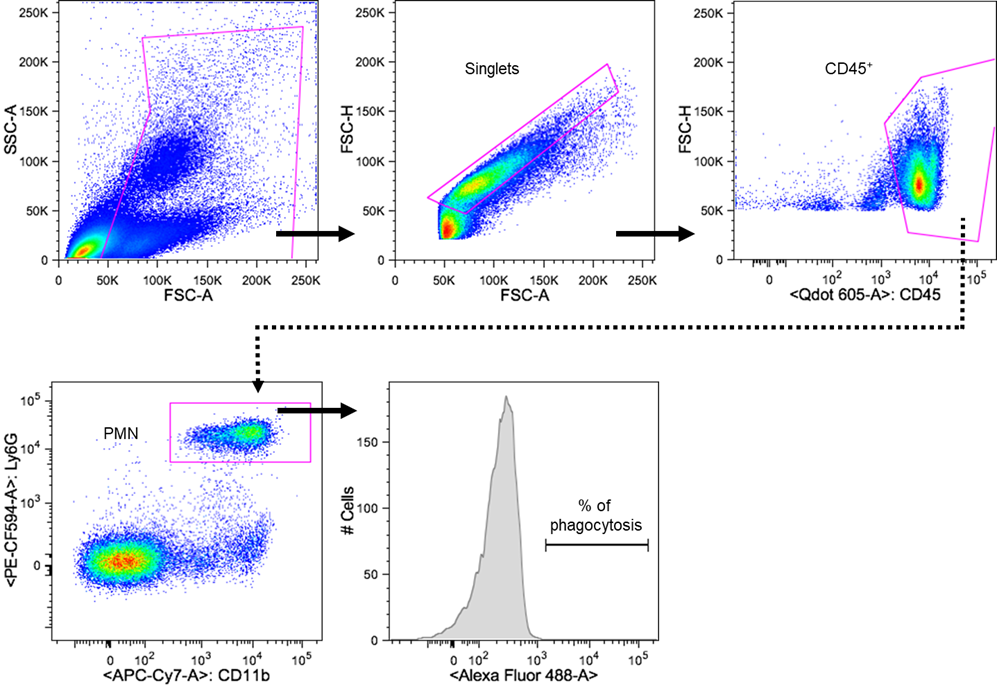
**

**Figure S1. Representative gating strategies used in this study. (A)** Gating strategy to analyze myeloid cell populations (neutrophils, monocytes, macrophages) in lung. **(B)** Gating strategy to analyze neutrophils in whole blood phagocytosis assay.

**Figure S2.** **Induction of pneumosepsis in wild-type mice.**

Induction of pneumonia and sepsis in C57BL/6 mice by intranasal inoculation with 10^4^ CFUs K. pneumoniae serotype 2 (ATCC 43816). CFU count in the lung **(A)** and spleen **(B)** at 6, 24, and 48 hours post-infection. The median is shown (n = 5 mice).

**Figure S3.** **Evaluation of pro-inflammatory cytokines and MPO in *K. pneumoniae* infection.**

Concentration of IL-1β, TNF-α, and MPO, in lung **(A, B, C)** and spleen **(D, E, F)** of wild-type mice after intranasal inoculation with 10^4^ CFUs K. pneumoniae. Each dot is mean ± SEM, (n = 4 mice). *P < 0.05 comparing with 24h and # P <0.05 comparing with 6 hours post-infection. Groups were compared using two-tailed Mann-Whitney test.

**
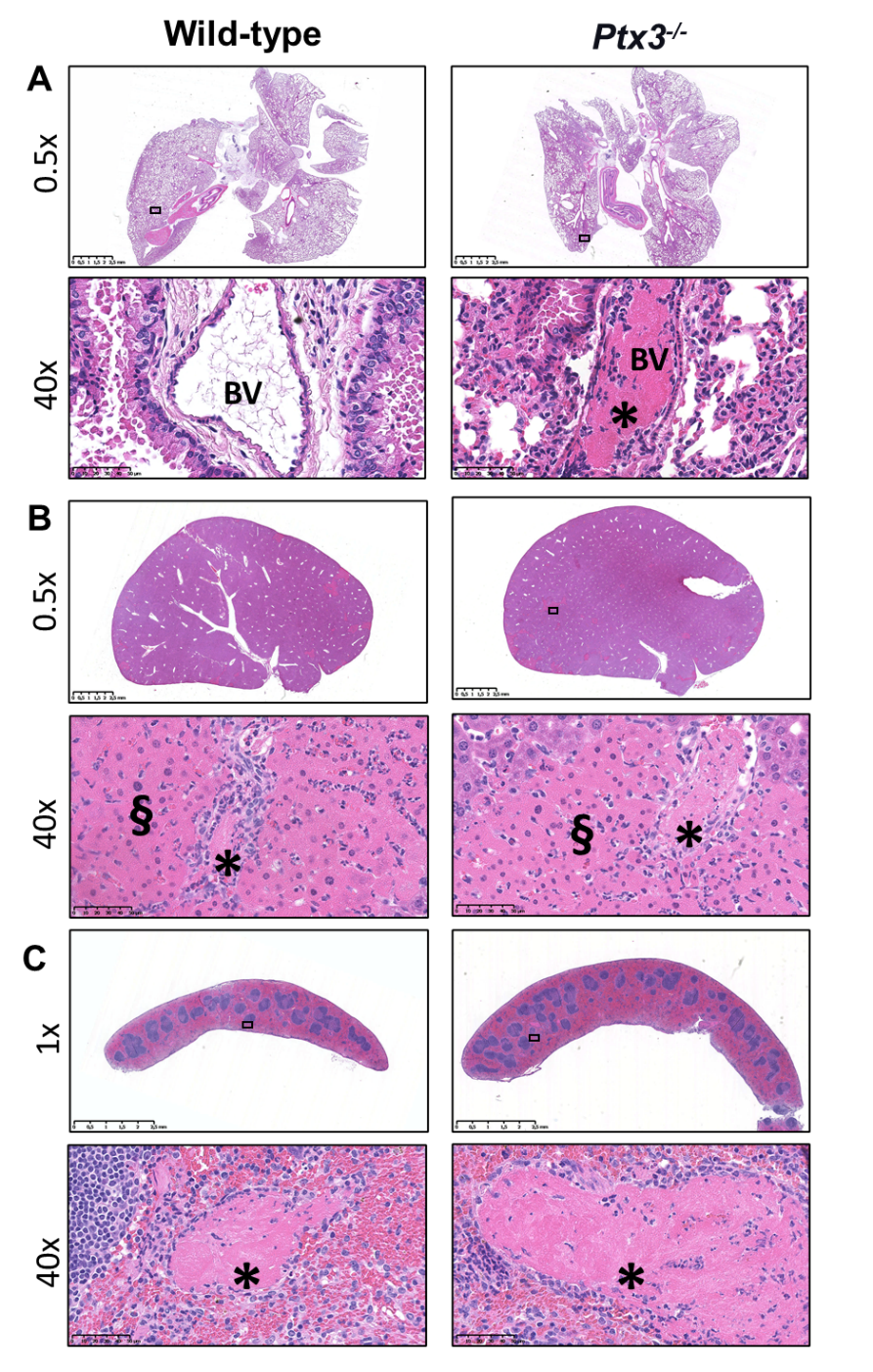
**

**D**

**E**

**F**

**IV fibrinocellular thrombi**

**Figure S4.** **Histological evaluation of lungs, liver, and spleen of mice at 24h after intraperitoneal K. pneumoniae infection**. Left (WT) and right (Ptx3^-/-^) panels show histological images of the lung **(A)**, liver **(B)** and spleen **(C)** with different magnifications. * = thrombosis, BV= blood vessel, § = liver coagulative necrosis. **D, E**, and **F**are the quantification of the number of IV fibrinocellular thrombi in the lung, liver, and spleen, respectively (n = 4-5 mice). Mean ± SEM is shown. The outlier was removed through ROUT. Groups were compared using two-tailed Mann-Whitney test.
